# Red and Processed Meat Consumption and Risk of Depression: A Systematic Review and Meta-Analysis

**DOI:** 10.3390/ijerph17186686

**Published:** 2020-09-14

**Authors:** Daniele Nucci, Cristina Fatigoni, Andrea Amerio, Anna Odone, Vincenza Gianfredi

**Affiliations:** 1 Digestive Endoscopy Unit, Veneto Institute of Oncology IOV-IRCCS, Via Gattamelata 64, 35128 Padua, Italy; dnucci.prof@gmail.com; 2 Department of Pharmaceutical Science, University of Perugia, Via del Giochetto, 06123 Perugia, Italy; cristina.fatigoni@unipg.it; 3 Department of Neuroscience, Rehabilitation, Ophthalmology, Genetics, Maternal and Child Health (DINOGMI), Section of Psychiatry, University of Genoa, 16132 Genoa, Italy; andrea.amerio@unige.it; 4 IRCCS Ospedale Policlinico San Martino, 16132 Genoa, Italy; 5 Department of Psychiatry, Tufts University, Medford, MA 02111, USA; 6 School of Medicine, Vita-Salute San Raffaele University, 20132 Milan, Italy; odone.anna@hsr.it; 7 CAPHRI Care and Public Health Research Institute, Maastricht University, 6211 Maastricht, The Netherlands

**Keywords:** depression, red meat, processed meat, meta-analysis

## Abstract

Depression is one of the leading causes of disability worldwide, with more than 264 million people affected. On average, depression first appears during the late teens to mid-20s as result of a complex interaction of social, psychological and biological factors. The aim of this systematic review with meta-analysis is to assess the association between red and processed meat intake and depression (both incident and prevalent). This systematic review was conducted according to the methods recommended by the Cochrane Collaboration and the Preferred Reporting Items for Systematic Reviews and Meta-Analyses guidelines. Relevant papers published through March 2020 were identified by searching the electronic databases MEDLINE, Embase and Scopus. All analyses were conducted using ProMeta3 software. A critical appraisal was conducted. Finally, 17 studies met the inclusion criteria. The overall effect size (ES) of depression for red and processed meat intake was 1.08 [(95% CI = 1.04; 1.12), *p*-value < 0.001], based on 241,738 participants. The results from our meta-analysis showed a significant association between red and processed meat intake and risk of depression. The presented synthesis will be useful for health professionals and policy makers to better consider the effect of diet on mental health status.

## 1. Introduction

### 1.1. Background

Depression is one of the leading causes of disability worldwide, with more than 264 million people affected [1]. One in six people (16.6%) experiences depression at some time in their life, more likely for women than men [2]. On average, depression first appears during the late teens to mid-20s as a result of a complex interaction of social, psychological and biological factors [3]. Depressive symptoms are often overlooked and untreated, and they are accompanied by poorer functioning compared to medical conditions [3,4]. Moreover, depression can increase the perception of poor health, the utilization of health care services and costs, as well as the burden on patients’ families and caregivers [5]. At its worst, depression can lead to suicide, the second leading cause of death in 15–29-year-olds [6].

A growing number of studies are focusing on the important role played by lifestyles and in particular diet, in both preventing and treating depression. The potential biological mechanisms underlying the association between diet and depression are still not completely understood. However, evidence in literature has pointed towards the involvement of food components in the monoamine synthesis, inflammation processes, hypothalamic–pituitary–adrenal axis (HPA) regulation, and neurogenesis [7]. Promising evidence currently focuses on the role of gut permeability and microbiota [8], and the interconnection between gut and brain [9]. Evidence has shown that dietary patterns are characterized by high intakes of fruit, vegetables, whole grains, fish and low intakes of red and processed meat could contribute to the prevention of depression, potentially due to their high content of antioxidants and folates [10], and probably due to the high content of long-chain omega-3 polyunsaturated fatty acids [11]. On the contrary, a higher consumption of refined and processed foods, as well as high-fat and high-sugar products, is associated with a higher risk of depression [12]. In particular, red and processed meats are rich in saturated fats, and a high consumption, which is typical in the so-called Western-diet, might be associated with pro-inflammatory states [13]. Evidence has shown that high levels of systemic inflammation and other factors, as for instance, the high levels of heme iron [14,15,16], the presence of exogenous N-nitroso compounds including nitrates and nitrites (especially for processed meat) [17,18], and the formation of polycyclic aromatic hydrocarbons and heterocyclic amines during cooking processes [19]—they are considered to be some of the main factors that increase the risk of cancer in high consumers of red and processed meat. According to the third World Cancer Research Fund and the American Institute for Cancer Research (WCRF/AICR) expert report published in 2018 [20], there is strong evidence that a high consumption of red and processed meat increases the risk of colorectal cancer, which is the third most commonly diagnosed cancer in males and the second in females (GLOBOCAN) worldwide [21]. In this context, it should also be considered that depression affects more than 10% of cancer patients, and presents a multifactorial pathogenesis involving psychosocial, biological and iatrogenic causes [22]. With regard to psychological and social causes, the negative effects of cancer diagnosis, prognosis and treatment on patients’ independence, abilities, family and economic status, can turn a subclinical sadness into a major depression [23]. From a biological point of view, many mechanisms seem to be implicated in the development of depression. High levels of systemic inflammation could increase the risk of several mental diseases, including depression [24]. Moreover, a high intake of saturated fatty acids seems to be associated with a lower level of brain derived neurotrophic factor (BDNF), neuroplasticity and cognitive ability [25], that are involved in the pathogenesis of depression [5,6]. However, results are not concordant, and some observational studies have highlighted a protective effect of low-moderate consumption of red meat [26,27], probably due to the high bio-availability of vitamin B12, folates and zinc [28,29]. Zinc stimulates the BDNF expression, promoting differentiation and plasticity; its deficiency, on the contrary, decreases neurogenesis and increases the risk of depressive symptoms development [30]. Vitamin B12 and folates are two of the most important coenzymes involved in the one-carbon metabolism, a metabolic pathway used to produce S-adenosylmethionine (SAM) [31]. SAM is a universal donor of methyl groups, largely implicated in several neurocognitive and neurological functions. A low availability of SAM is associated with higher depressive tendencies [32].

Considering that (i) studies’ results are not concordant; (ii) biological mechanisms behind red and processed meat intake and risk of depression are not completely known, and (iii) the important role of diet in preventing several chronic diseases, including mental diseases (such as depression), we conducted a systematic review and meta-analysis exploring the association between red and processed meat intake and the risk of depression.

### 1.2. Aim of the Study

The aims of the current systematic review with meta-analysis are: first to collect and retrieve previous studies focusing on red and processed meat intake and depression; and second, to estimate the strength of association between red and processed meat consumption and depression (both incident and prevalent).

## 2. Materials and Methods

The following systematic review and meta-analysis was conducted according to the methods recommended by the Cochrane Collaboration [33] and to the Meta-analysis Of Observational Studies in Epidemiology (MOOSE) guidelines [34], and the process and results were documented according to the Preferred Reporting Items for Systematic Reviews and Meta-Analyses [35] guidelines [36].

### 2.1. Information Sources and Search Strategy

In order to perform the structured computer literature search, the following databases were searched: PubMed/Medline, Excerpta Medica dataBASE (EMBASE) and Scopus. The literature search was conducted using a pre-determined combination of keywords, according to the type of database consulted. When possible, medical subject headings (MeSH or similar) or free text words were used. The keywords were selected considering two features: depression, and red and/or processed meat consumption. Then, the selected keywords were combined using Boolean operators AND/OR and NOT. The strategy was first developed in PubMed/Medline and then adapted for use in the other databases (Supplementary Table S1). The literature search was carried out in March 2020. In addition, further studies were retrieved from reference listing of relevant articles and consultation with experts in the field.

### 2.2. Inclusion and Exclusion Criteria

In order to be included in the systematic review, the collected references had to report the results of the primary research evaluating the association between depression (remission or relapse), either as a continuous or binary variable, in adult men and women, and the intake of red and/or processed meat. Both population-based and hospital-based studies were included. Among hospital-based studies, inpatients, day-hospital, and outpatient subjects were included, while emergency care records were excluded, as they were considered non-representative. Other restriction criteria were: had to be written in English and provide full text. No time filter was applied; however, non-human studies (animal models), non-original papers (e.g., reviews, book chapter, letters to the editor, brief note, commentaries, conference paper) were excluded. Table 1 shows a detailed description of inclusion/exclusion criteria according to the Population, Exposure, Outcomes and Study design (PEOS) [37], adjusted for observational studies extended with time and language filters, as recommended by the Cochrane Collaboration [38].

### 2.3. Data Extraction

A two-step process was adopted to identify relevant articles. Two authors (DN and CF) independently screened title and abstract of the retrieved publications, to collect potentially relevant articles. The full text was obtained only for selected papers, based on compliance with inclusion/exclusion criteria. Data were extracted from included studies, by two authors in blind (DN and CF), using a pre-defined spreadsheet. The spreadsheet was elaborated in Microsoft Excel^®^ for Windows (Redmond, WA, USA, 2007) and was pre-piloted, on 10 randomly selected papers, to ensure methodological concordance among the Authors. As done before [39,40,41,42], the spreadsheet was used to systematically record qualitative and quantitative data extracted from the included studies. Quantitative data recorded included: age, sample size, depressed subjects, loss of follow-up, red and processed meat intake. For each included study, the name of the first author, year of publication, and the original country where the study was conducted were collected. In case of incomplete available data, the corresponding authors were contacted by e-mail. Any disagreement in both article screening and data extraction was solved through discussion between the two researchers. If the disagreement persisted, a third researcher was consulted (VG).

### 2.4. Quality Evaluation

The quality evaluation of the included publications was independently assessed by two Authors using the New–Ottawa Scale [43]. If disagreement was found among researchers, it was solved through discussion. The New–Ottawa Scale refers to three potential risks of bias: selection of participants, comparability, and outcomes/exposure. The total score can range from 0 (the poorest quality) to 10 (the highest quality). The quality assessment score was calculated for each study and tabulated with the other characteristics extracted from the studies.

### 2.5. Meta-Analysis

Individual study data were pooled using ProMeta3^®^ (Internovi, Italy) software. Fixed and random effects were used in this study according to the heterogeneity. Fixed effect presumes that there is one equal true exposure effect for all the studies, whereas the random-effect model presumes that the true exposure effect in any of the analyzed studies may be different in each study [44]. Based on this assumption, it is commonly accepted to use a random effect model if heterogeneity is high. The heterogeneity was estimated through Chi^2^ and I^2^ tests. Values of I^2^ above 75% are classified as high heterogeneity, values between 50–75% are classified as moderate heterogeneity, values between 25–50% are classified as low heterogeneity, while below 25% as no heterogeneity. The pooled effect size (ES) was calculated as odds ratio (OR) and its relative 95% confidence interval (95% CI). We assessed publication bias with the visual inspection of a funnel plot [33] and the Begg [45] and Egger tests [46]. Statistical significance was set at *p* < 0.10 [46]. A “trim and fill” method was used if publication bias was detected [47]. The “trim and fill” method is a statistical approach used to adjust for publication bias [48]. It is aimed to estimate potential missing studies, causing the asymmetry of the funnel plot [49]. This method assumes that the studies with the most extreme ES have to be suppressed, adjusting the overall effect estimate [50].

### 2.6. Subgroup and Sensitivity Analysis

In order to exclude the potential overlapping effect due to the inclusion of datasets reporting results for different levels of outcome (minor and major depression; depression and subsyndromal depression) using the same pool of subjects, a sensitivity analysis was performed, excluding these data [51,52]. If there were three or more studies with relevant data, subgroup analyses were planned. In particular, four additional sensitivity analyses were conducted, considering the following: (i) study design, (ii) including only studies using a validated tool to assess meat intake, (iii) including only studies using a validated tool to diagnose depression, (iv) if QS was ≥8. Moreover, a subgroup analysis by gender was conducted in order to estimate potential different effects among the two groups.

## 3. Results

### 3.1. Literature Search

**Figure 1:**
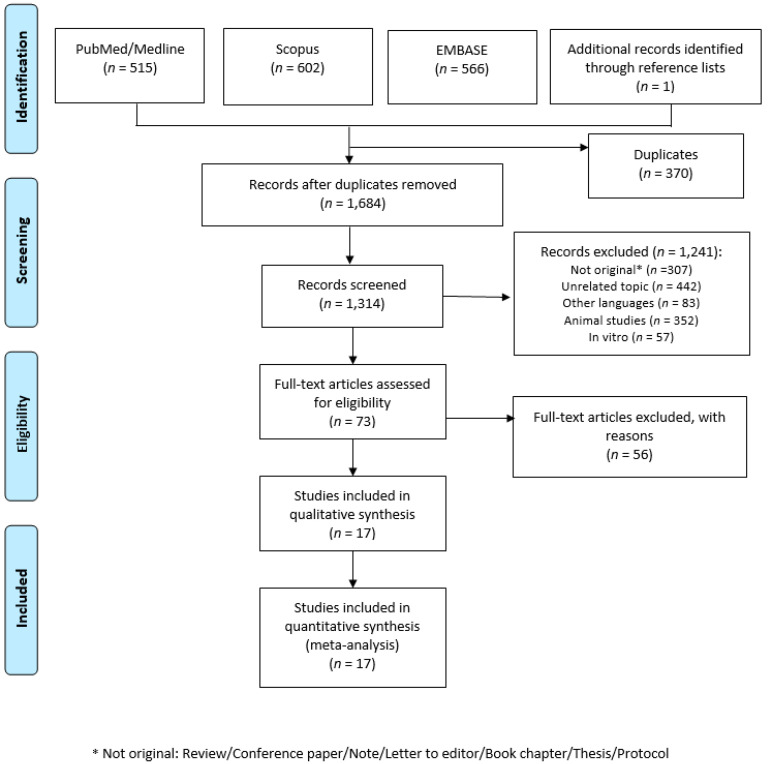
Flow diagram of the selection process. From: Moher D, Liberati A, Tetzlaff J, Altman DG, The PRISMA Group (2009). Preferred Reporting Items for Systematic Reviews and Meta-Analyses: The PRISMA Statement. PLoS Med 6(7): e1000097. doi:10.1371/journal.pmed1000097 [35].

A total of 1684 articles were retrieved. After a preliminary screening, 370 articles were excluded because they were duplicates, 307 were not original papers (review, letter to editor, editorial, protocols, etc.), and 442 covered different topics. After title and abstract screening, a total of 73 articles were consulted in full, while at the end of the screening procedure, 17 articles were included in the systematic review [26,27,53,54,55,56,57,58,59,60,61,62,63,64,65,66,67]. Figure 1 shows the selection process. One longitudinal study reported separate data for baseline and follow-up, and for this reason it was considered separately [65]. Moreover, two studies reported separate data for men and women, and for this reason they were considered separately [26,55]. Furthermore, Barros et al. reported separate data for minor and major depression [54], and Goh reported separate data for depression and subsyndromal depression, and for this reason they were considered separately [58]. Lastly, Won et al., reported data stratified by age groups (19–29; 30–49; 50–64 years), and for this reason, it was considered separately [67], resulting in 24 datasets being included in the meta-analysis.

### 3.2. Characteristics of the Included Studies

Characteristics of the included studies are reported in Table 2. Seven studies were performed in Asia [27,53,58,62,63,64,67], six studies were conducted in Europe [26,55,56,57,59,66], two studies were conducted in the Americas [54,61], and two studies were conducted in Australia [60,65]. The first cross-sectional [26] and longitudinal [66] studies assessing red and processed meat intake and risk of depression were published in 2009. Most of the studies were cross-sectional studies (*n* = 9), followed by longitudinal (*n* = 5, of which one reported also cross-sectional analysis), and case-control (*n* = 3). Ten studies used a Food Frequency Questionnaire (FFQ) to assess red and processed meat intake, whilst one study used the 24-h dietary recall interviews performed by trained interviewers [61]. However, seven studies did not use a validated tool to assess the meat intake, or did not specify whether the adopted questionnaire had been previously validated or not. Regarding depression, the tools used to make the diagnosis were heterogeneous (as, for instance, Patient Health Questionnaire-9 (PHQ-9), Beck Depression Inventory [58], Center for Epidemiologic Studies Depression Scale (CESD)). Most of the time, PHQ-9 (*n* = 4) and BDI (*n* = 3) were used; however, almost all the studies used a validated tool (*n* = 15/17). Lastly, the results were expressed using different measures, as, for instance, odds ratio (OR), hazard ratio (HR), β coefficient (β), and Spearman’s rho (r). Regarding the quality assessment, the score ranged between 5 and 10.

### 3.3. Characteristics of the Studied Populations

The smallest sample size included in a study was 46 participants [53], whereas the largest study size was 49,025 participants [54]. The age of the subjects was reported as mean and SD in the majority of the study, while in five studies, the age range was recorded [53,58,60,61,67], and in one study, the subjects’ ages were not available [66]. The age of the participants ranged from 18–93 years. Subjects were randomly selected from the population (or a subgroup, as students in 3 [26,55,66]) in all studies, except for two studies, where they were psychiatric patients treated in a psychiatric clinic [62,64]. All studies included both men and women, but in four studies, only women were included [53,60,65,67]. However, in two studies, Authors reported the results for men and women separately. Red and processed meat intake was reported using different units in the original studies (i.e., g/day, g/kg per day or frequency per week or day), limiting the comparability of the intake.

### 3.4. Results of Meta-Analysis

**Figure 2:**
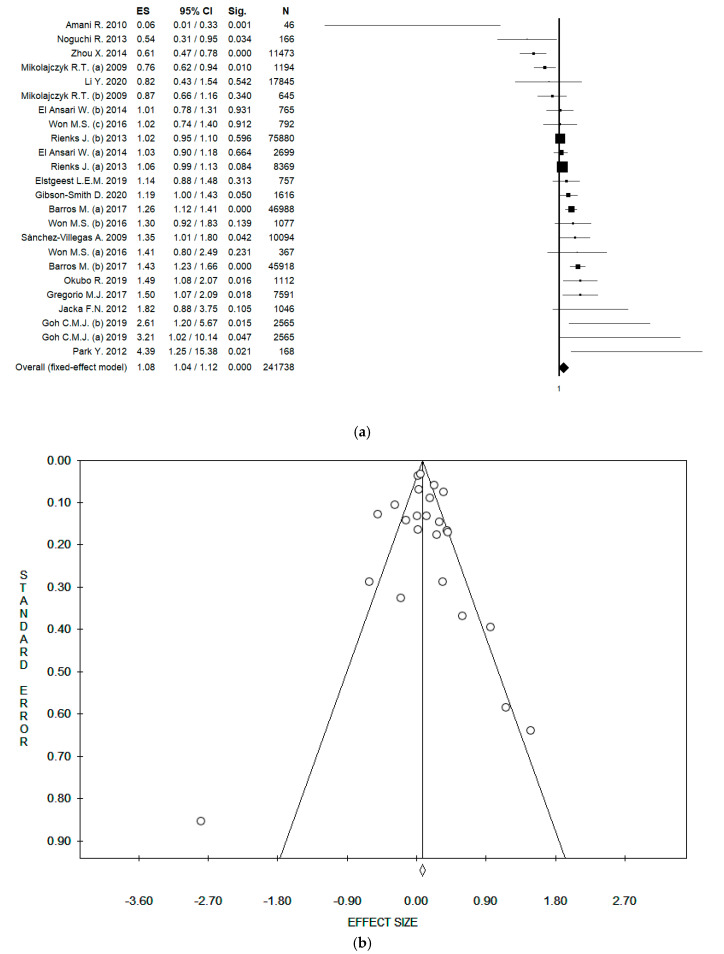
(**a**) Forest plot, and (**b**) funnel plot of the meta-analysis assessing the association between meat intake and depression. ES, effect size; CI, confidence interval.

Considering all the 24 datasets, and using the fixed effect model, the pooled ES was 1.08 [(95% CI = 1.04; 1.12), *p*-value < 0.001] (Figure 2a); while using the random effect model, the pooled ES was 1.10 [(95% CI = 1.00; 1.22), *p*-value = 0.055] based on 241,738 participants with high statistical heterogeneity (Chi^2^ = 102.59, df = 23, I^2^ = 77.58, *p*-value < 0.001). No potential publication bias was found by the visual assessment of the funnel plot (Figure 2b), and this was confirmed by the Egger’s linear regression test (intercept 0.32, t = 0.48, *p*-value = 0.639).

### 3.5. Sensitivity Analysis

In order to estimate the effect of meat intake without potential overlapping effects, two datasets (assessing minor depression [54] and subsyndromal depression [58]) were excluded. Using the fixed effect model the pooled ES was 1.06 [(95% CI = 1.02; 1.10), *p*-value = 0.002], while using the random effect model, the pooled ES was 1.08 [(95% CI = 0.97; 1.29), *p*-value = 0.166], based on 192,185 participants with high statistical heterogeneity (Chi^2^ = 89.90, df = 21, I^2^ = 76.64, *p*-value < 0.001). No potential publication bias was found by the visual assessment of the funnel plot and confirmed by the Egger’s linear regression test (intercept 0.20, t = 0.29, *p*-value = 0.777).

**Figure 3:**
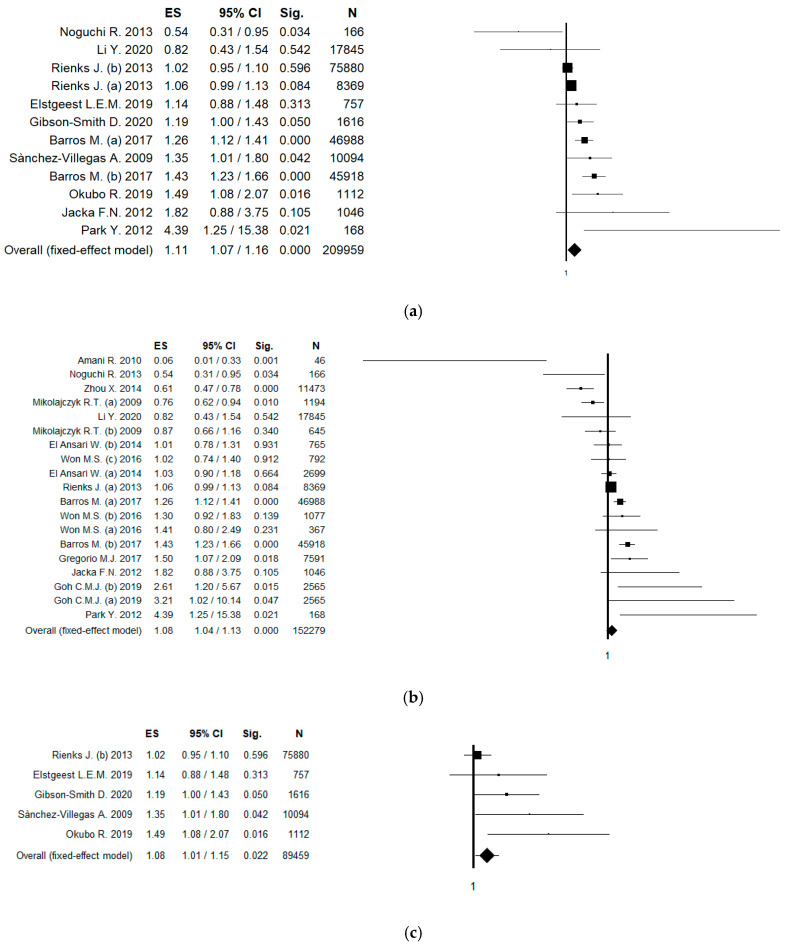
(**a**) Forest plot of the meta-analysis assessing the association between meat intake and depression, only including studies that used validated tools to assess meat intake; (**b**) forest plot and meta-analysis assessing the association between meat intake and prevalent depression (only including case-control and cross-sectional studies); (**c**) forest plot and meta-analysis assessing the association between meat intake and incident depression (only including longitudinal studies). ES, effect size; CI, confidence interval.

In order to increase the robustness of results, a sensitivity analysis only including studies that used validated tools to assess meat intake was conducted. In this analysis, 10 studies (12 datasets) were included, and the pooled ES was 1.11 [(95% CI = 1.07; 1.16), *p*-value < 0.001] in the fixed effect model (Figure 3a); while using the random effect model, the pooled ES was 1.18 [(95% CI = 1.06; 1.32), *p*-value = 0.002], based on 209,959 participants with moderate statistical heterogeneity (Chi^2^ = 41.70, df = 11, I^2^ = 73.62, *p*-value < 0.001). No potential publication bias was found by the visual assessment of the funnel plot and confirmed by Egger’s linear regression test (Intercept 1.17, t = 1.39, *p*-value = 0.194). A sensitivity analysis only including studies that used validated tool to diagnose depression was performed. In this analysis, 15 studies (20 datasets) were included, and the pooled ES was 1.07 [(95% CI = 1.04; 1.12), *p*-value < 0.001] in the fixed effect model; while using the random effect model, the pooled ES was 1.08 [(95% CI = 0.97; 1.20), *p*-value = 0.175], based on 228,250 participants with high statistical heterogeneity (Chi^2^ = 98.14, df = 19, I^2^ = 80.64, *p*-value < 0.001). No potential publication bias was found by the visual assessment of the funnel plot, but a border-line statistically significant publication bias was found by the Egger’s linear regression test (Intercept 0.15, t = 0.18, *p*-value = 0.857).

In order to differentiate among the risk of prevalent and incident depression, a sensitivity analysis based on study design was conducted. Referring to prevalent depression, only case-control and cross-sectional studies were included. In this case, 12 studies (19 datasets) were included, and using the fixed effect model, the pooled ES was 1.08 [(95% CI = 1.04; 1.13), *p*-value < 0.001] (Figure 3b); while using the random effect model, the pooled ES was 1.07 [(95% CI = 0.94; 1.23), *p*-value = 0.317], based on 152,279 participants with high statistical heterogeneity (Chi^2^ = 92.71, df = 18, I^2^ = 80.58, *p*-value < 0.001). No potential publication bias was found by the visual assessment of the funnel plot and confirmed by the Egger’s linear regression test (intercept −0.06, t = −0.07, *p*-value = 0.946). Referring to incident depression, only cohort (longitudinal) studies were included. In this case, five studies (five datasets) were included, and using the fixed effect model, the pooled ES was 1.08 [(95% CI = 1.01; 1.15), *p*-value = 0.022] (Figure 3c); while using the random effect model, the pooled ES was 1.18 [(95% CI = 1.02; 1.35), *p*-value = 0.023], based on 89,459 participants with low statistical heterogeneity (Chi^2^ = 9.85, df = 4, I^2^ = 59.39, *p*-value = 0.043). A potential publication bias was found by the visual assessment of the funnel plot and confirmed by the Egger’s linear regression test (intercept 2.39, t = 4.98, *p*-value = 0.016). The estimated ES slightly changed after the “trim and fill” method was applied (ES = 1.03 [(95% CI = 0.97; 1.09), *p*-value = 0.332; ES was 1.04 [(95% CI = 0.91; 1.19), *p*-value = 0.568 for fixed and random effect, respectively).

Lastly, a sensitivity analysis only including studies with QS ≥8 was conducted, for a total of 12 studies (15 datasets). Using the fixed effect model, the pooled ES was 1.10 [(95% CI = 1.05; 1.14), *p*-value = 0.001]; while using the random effect model, the pooled ES was 1.14 [(95% CI = 1.02; 1.26), *p*-value = 0.018], based on 219,389 participants with high statistical heterogeneity (Chi^2^ = 59.907 df = 14, I^2^ = 76.66, *p*-value < 0.001). No potential publication bias was found by the visual assessment of the funnel plot and confirmed by the Egger’s linear regression test (Intercept 0.74, t = 0.86, *p*-value = 0.405). Results are summarized in Table 3.

### 3.6. Subgroup Analysis by Gender

The sub-group analysis considering only women, included six studies (nine datasets), and the pooled ES was 1.03 [(95% CI = 0.99; 1.08), *p*-value = 0.171]; while using the random effect model, the pooled ES was 1.02 [(95% CI = 0.91; 1.14), *p*-value = 0.724], based on 91,470 participants with moderate statistical heterogeneity (Chi^2^ = 25.14, df = 8, I^2^ = 68.17, *p*-value = 0.001). No potential publication bias was found by the visual assessment of the funnel plot and confirmed by the Egger’s linear regression test (intercept −0.45, t = 0.0–0.489, *p*-value = 0.645).

## 4. Discussion

The current paper analyzes data from a large and systematic review with meta-analysis, conducted using three medical-scientific databases (PubMed/Medline, EMBASE and Scopus). Out of 1684 retrieved articles, 17 studies were included in the quantitative and qualitative analysis; however, because some of them reported data separately (for level of depression and gender), the whole sample was based on 24 databases. The original studies included were mainly conducted in Asia, and in most of the cases, they had a cross-sectional design. The pooled data obtained from this meta-analysis suggest that red and processed meat intake might potentially be a risk factor for depression, with a small but significant increment of depression risk (ES = 1.08 [(95% CI = 1.04; 1.12), *p*-value < 0.001], based on 241,738 participants). However, the association was attenuated when the random effect model was applied, with a weak boarder-line statistical significance [ES = 1.10 [(95% CI = 1.00; 1.22), *p*-value = 0.055].

To better understand the strength of the association and to assess the robustness of our results, several sensitivity analyses have been performed: firstly removing potential overlapping data, that did not materially change the results, secondly, we only included studies that used validated tools (to assess meat intake and to diagnose depression), finding a higher strength of the association and lower heterogeneity; thirdly, we differentiated among prevalent and incident depression, confirming the consistency of our results especially for incident depression. However, it should be considered that only a small number of longitudinal studies (only five) have been retrieved. Moreover, cohort studies are more susceptible to selection bias, and maintaining a follow up might be problematic, particularly among depressed individuals, who might be less prone to taking part in the study. On the contrary, considering the natural history of depression, a chronic and remittent disease with a long latency, it could be better evaluated by employing a case-control or cross-sectional study design. However, case-control and cross-sectional studies are notoriously susceptible to potential recall bias; nevertheless, the high number of participants included, the high number of retrieved studies and the use of validated tools in most of the included studies might reduce this disadvantage. Lastly, a sensitivity analysis only including studies with QS ≥ 8 was conducted. In this case, the ES for both fixed and random effects was strongly significant, confirming the robustness of our results.

Original studies assessed the exposure (red and processed meat intake) mainly through validated food frequency questionnaires. FFQ is a cheap and manageable tool, largely used to measure dietary intake; nevertheless, it cannot be considered free of potential bias (both under- and over-estimation). Moreover, since the FFQs used in the original studies were different, the intake was reported using various units of measures and quantities (i.e., portion per week/day or g/day). For that reason, we could not perform a dose–response analysis. Furthermore, the quantity of meat intake largely differs among studies, potentially limiting the comparability of the data. This aspect might partially explain the moderate-to-high heterogeneity found in our meta-analysis. Because of the above-mentioned factors, the included studies reported mixed findings on the association between meat intake and risk of depression, which could explain the slight differences in ES obtained using fixed and random effect models.

The subgroup analysis by gender was only possible for women, since only two studies reported data for men. When only women were taken into account, the ES was not statistically significant anymore, even if the direction of the association was confirmed. The absence of statistical significance might be truly due to a lack of association between meat intake and depression among women, or it could be due to the lower number of participants; as a matter of fact, the analysis carried out among women involved a total of 91,470 subjects, compared to 241,738 participants considering both men and women.

On the whole, our effect sizes were small, indicating that, even if the association between meat intake and depression was significant, the impact of meat on depression was small at an individual level; however, it could be of clinical importance at the population level. To the best of our knowledge, this represents the first systematic and meta-analytic study evaluating the association between red and processed meat and the risk of depression. Actually, a previous meta-analysis conducted by Zhang et al. in 2017 focused on meat in general [68]. As a matter of fact, they included studies that did not differentiate among red and processed meat or poultry; furthermore, only eight studies were included, and studies including adolescent and pregnant women were also considered eligible. Moreover, they did not perform sensitivity analysis or subgroup analysis by gender.

Our results are particularly relevant considering that depression is one of the primary causes of disease burden all over the world. In the last decades, a growing body of evidence was built to identify potential lifestyle factors associated with depression, and great attention has been posed, particularly on diet and depression. Even if the potential biological mechanisms behind the association between red/processed meat and depression, our results seem to confirm that foods rich in fats (especially reach in saturated fatty acids) and processed food (such as, for instance, red and processed meat) are associated with an altered HPA [69]. Moreover, it should be considered that a high intake of fatty and processed foods is correlated with a pro-inflammatory activity, causing a detrimental effect on the cardiovascular system [70], increasing the risk of depression (if the microvascular dysfunction is located in the brain) [12].

Nevertheless, it should be considered that people who have a healthy dietary approach are usually accustomed to adopt other healthy behaviors, such as being physically active and avoiding smoking [71,72]. This is because these decisions are based on the same decision-making model, and in particular diet and physical activity share the same interconnected factors [73]. Moreover, it should be considered that both diet and physical activity play a synergic effect on body composition, which in turn seems to be associated with depression [74]. Lastly, according to a recent meta-analysis, physical activity is significantly associated with a lower risk of both prevalent and incident depression, representing a potentially useful intervention to prevent and treat depression [75]. Some of the original studies included in the current meta-analysis also considered the level of physical activity performed as a potential confounder (in the adjusted model). This could be another potential explanation for the mixed results found in literature and for the high heterogeneity obtained in our meta-analysis. However, a high I^2^ value implies that heterogeneity is straightly due to heterogeneity amongst studies, rather than a sampling error [76].

However, the results from our review found a marginal but significant detrimental effect of red and processed meat on depression. In this regard, nutritional education campaigns should be promoted [77,78,79], especially because adherence to a healthy diet among the adult population is still low [80,81].

### Limitantions and Strengths

The main limitation of our study is the high I^2^ value that might limit the generalizability of our results. However, with the aim of reducing heterogeneity, we performed several sensitivity analyses obtaining a moderate and low heterogeneity when only studies adopting a validated tool to evaluate meat intake, and only longitudinal studies respectively, were considered. Moreover, the high number of sensitivity analyses performed increased the robustness of our study, since the results did not considerably change. The mixed findings reported by each included study could be due to the different tool used to assess meat intake or to the different portion considered. Moreover, for the above-mentioned reasons, it was neither feasible to conduct a dose-response analysis, nor to identify a recommended intake of meat. The main strengths of this review are: being the first systematic review with meta-analysis aiming to assess the association between red and processed meat and risk of depression; being systematic in nature, as well as the comprehensive approach used to retrieve as much evidence as possible by consulting three different medical-scientific databases, and by manually checking the listed references; have been conducted according to the Cochrane Collaboration and MOOSE guidelines, and documented according to the PRISMA guidelines. Furthermore, the sample size was particularly large, based on 241,738 participants; moreover, a subgroup analyses by sex has been conducted as well. In addition, based on study design we were able to estimate the ES for both prevalent and incident depression. Moreover, a variety of confounding variables were chosen in the original studies and, in order to control the results, we combined data with the highest level of adjustment.

## 5. Conclusions

To conclude, the results of this systematic review and meta-analysis show a statistically significant detrimental effect of red and processed meat intake on depression (mainly prevalent). This study is important because our results might be used by clinicians and policy makers (considering the impact at the population level of our results) to design and implement interventions aiming to reduce the burden of depression in our society. However, it should be considered that our effect sizes were small, based on very mixed findings reported by each included study, and with high heterogeneity. For these reasons, further studies are needed. Consortium studies should be encouraged in order to harmonize data collection methods and results presentation.

## Figures and Tables

**Table 1:** Detailed description of inclusion/exclusion criteria according to a Population, Exposure, Outcomes and Study design (PEOS).

Search Strategy	Details
Inclusion criteria	P: adults (men and women)
	E: high intake of red and processed meat
	O: Depressive disorder
	S: cohort studies, case-control, cross-sectional
Exclusion criteria	P: people < 18 years old, pregnant women, patients with chronic diseases
	E: combined consumption of multiple food components (e.g., dietary pattern)
	O: other psychological disorders
	S: not original papers (opinion paper, review article, commentary, letter, protocols, article without quantitative data, thesis, conference papers, note, book chapter), trials
Language filter	English
Time filter	No filter (from inception)
Database	PubMed/Medline; EMBASE, Scopus

**Table 2:** Descriptive characteristics of the included studies listed in alphabetical order.

Author, Year [Reference]	Country	Study Design	Study Period	Sample Size, Gender, Age	N. Depressed Subjects, Gender, Age	Attrition+	Diagnosis of Depression	Validated Tool, for Depression	Tool used to Assess Meat Intake	Validated Tool, for Meat Intake	Portion of Meat	OR, HR, β, r (CI95%)	Adjustment	QS
Amani R., 2010 [53]	Iran	case-control	2006	46 F, range 20–25 y	23 mean age: 20.7 ± 1.6 y	262	21-BDI	yes	12-item semiquantitative FFQ	n.a.	3–4 times/week	2 cases/14 controls *p* < 0.001	crude model	7
Barros M., 2017 [54]	Brazil	cross-sectional	2013–2014	49,025 (25,542 F; 23,483 M) mean: 37 y	3107 minor depression; 2037 major depression	11,177 (M; F)	PHQ-9	yes	12-item FFQ	yes	weekly consumption	Minor depression: OR 1.26 (1.12–1.41)	age, sex, and education	9
												Major depression: OR 1.43 (1.23–1.66)		
El Ansari W., 2014 [55]	England, Walls, Ireland	cross-sectional	2007–2008	3464 (2699 F; 765 M) mean: 24.9 ±8.6	n.a.	242 (M; F)	20-items Modification of BDI	yes	12-item questionnaire	no	several times a day	F: r = 0.008 *p* = 0.680	crude model	7
												M: r = −0.003 *p* = 0.942		
Elstgeest L.E.M., 2019 [56]	Italy	longitudinal study 3-y FU	1998–2009	baseline 1058 (579 F; 479 M) mean: 65.8 ±15.2 FU1: 960, FU2: 853, FU3: 757	baseline: 187; FU1: 231; FU2: 145; FU3: 150	148 (M; F)	CES-D	yes	240-FFQ	yes	Quartiles of standardized intake	β = −0.39 (−1.13, 0.36) *p* = 0.313	baseline CES-D score, age, sex, marital status, education, PA, smoking, living disabilities, alcohol intake and energy intake.	10
Gibson-Smith D., 2020 [57]	the Netherlands	longitudinal 9-y FU	2004	1634 (1108 F; 526 M = mean: 52.0 ± 13.2	414 depressed and 886 remission	435 (M; F)	IDS-SR	yes	258-FFQ	yes	n.a.	β = −0.05 (−0.11, −0.00) *p* = 0.05	age, sex, education, marital status, PA, smoking status	9
Goh C.M.J., 2019 [58]	Singapore	cross-sectional	2013	2565 (1448 F; 1117 M) range: 60–85 y	425 subsyndromal; 177 depression	0	GMS-AGECAT	yes	National survey	no	n.a.	Depression: OR = 3.21 (1.02 10.14) *p* = 0.05	crude model	7
												Subsyndromal OR = 2.61 (1.20 5.67) *p* = 0.02		
Gregorio M.J., 2017 [59]	Portugal	cross-sectional	2013–2015	7591 (4784 F; 2807 M) mean: 48.02 ± 18.02	n.a.	2562 (M; F)	HADS	yes	food questionnaire not further specified	n.a.	n.a.	OR = 1.50 (1.07 2.09) *p* = 0.018	age, sex, education, employment, territorial units, smoking, PA and alcohol habits.	9
Jacka F.N., 2012 [60]	Australia	cross-sectional	2009	1046 F range: 20–93 y	60	81	SCID-I/NP	yes	Cancer Council dietary questionnaire	yes	>57 g/day	OR = 1.82 (0.88 3.75)	age and dietary pattern score	9
Li Y., 2020 [61]	USA	cross-sectional	2007–2014	17,845 (9102 F; 8743 M) range: 18–65 y	1647; (1070 F; 577 M)	0	PHQ-9	yes	24-h dietary recall interviews by trained interviewers	yes	0.20 g/kg per day	OR = 0.82 (0.43–1.54)	age, sex, race, marital status, education, income, BMI, diabetes, hypertension, smoking, alcohol, energy intake, fruit intake, vegetable intake, Mg intake, Zn intake, SFA intake, MUFA intake, PUFA intake and PA	10
Mikolajczyk R.T., 2009 [26]	Germany, Poland-Bulgaria	cross-sectional	2005	1839 (1194 F; 645 M), mean: 20.6 ± 2.3	n.a.	264 (M; F)	M-BDI	yes	12-item FFQ	no	n.a.	F: r = −1.38 *p* = 0.01	country and all the other food components	8
												M: r = −0.66 *p* = 0.34		
Noguchi R., 2013 [62]	Japan	cross-sectional	n.a.	166 (62 F; 104 M) mean: 38.7 ± 10.2	75 (25 F; 50 M)	0	H-SDS	yes	BDHQ-56 foods	yes	n.a.	r = −0.159	age, BMI and sex	8
Okubo R., 2019 [63]	Japan	longitudinal 5 y FU	1990–2014	1112 (652 F; 460 M) mean: 73 y	85	11,107	PHQ-9	yes	147-FFQ	yes	4 times/week	27 cases/244 controls	crude model	8
Park Y., 2012 [64]	Korea	case-control	2008–2010	168 (112 F; 56 M) mean: 44.85 ± 1.77 y	80 (59 F; 21 M)	0	CES	yes	91-FFQ	yes	>3.61 serving/week	OR = 4.39 (1.25–15.38)	Drinking, marital status, sleeping hours, education, job and energy except for energy intake	8
Rienks J., 2013 [65]	Australia	cross-sectional and longitudinal analysis 3-years FU	2001–2004	8369 F in cross-sectional; mean: 52.5 ± 1.5	721 in cross-section; 660 in longitudinal	2857	CES	yes	101-FFQ	yes	n.a.	OR = 1.06 (0.99–1.13) *p* = 0.11;	energy, smoking, PA, ability to manage on available income, occupation status, education, marital status, mean stress score and BMI	10
				7588 in longitudinal mean: 52.5 ± 1.5								HR = 1.02 (0.95–1.10) *p* = 0.54		
Sànchez-Villegas A., 2009 [66]	Spain	longitudinal 4.4 y FU	1999–2005	10,094 (F and M) age n.a.	480 (156 M, 324 F)	5347	self-reported	no	136-FFQ	yes	177 g/d M; 167 g/d F	HR= 1.35 (1.01–1.80)	sex, age, smoking, BMI, PA, energy intake, and employment	8
Won M.S., 2016 [67]	Korea	case-control	2013	2236 F range: 19–64 y	315	430	self-reported	no	112-FFQ	n.a.	0.20 ± 0.02 servings/day	19–29 y: 45 cases/322 controls; 30–49 y: 119 cases/958 controls; 50–64 y: 151 cases/641 controls	crude model	5
Zhou X., 2014 [27]	China	cross-sectional	2012–2013	11,473 (6155 F; 5318 M) mean: 53.72	n.a.	0	PHQ-9	yes	food questionnaire not further specified	n.a.	≥500 g/week	OR 0.61 (0.47–0.78)	crude model	7

F: female; M: male; y = years; FU: follow-up; N: number; n.a.: not available; BMI: Body Mass Index; PA: physical activity; FFQ: Food Frequency Questionnaire; Zn: Zinc; Mg: Magnesium; PUFA: Poly-unsaturated Fatty Acids; MUFA: mono-unsaturated acids; SFA: saturated fatty acids; BDI: Beck Depression Inventor; CES-D: Center for Epidemiologic Studies Depression scale; GMS-AGECAT: Geriatric Mental State with Automated Geriatric Examination for Computer Assisted Taxonomy; H-SDS: Himorogi Self-rating Depression Scale; HADS: The Hospital Anxiety and Depression Scale; IDS-SR: Inventory of Depressive Symptomatology—Self Report; PHQ-9: Patient Health Questionnaire–9; SCID-I/NP: The Structured Clinical Interview for DSM-IV-TR Research Version, non-patient edition.

**Table 3:** Results of sensitivity and subgroup analyses.

Analysis	N. of Participants	ES (95% CI)
without potential overlapping effect	192,185	Fixed effect: 1.06 (1.02; 1.10)
		Random effect: 1.08 (0.97; 1.29)
validated tool to assess meat intake	209,959	Fixed effect: 1.11 (1.07; 1.16)
		Random effect: 1.18 (1.06; 1.32)
validated tool to diagnosis depression	228,250	Fixed effect: 1.07 (1.04; 1.12)
		Random effect: 1.08 (0.97; 1.20)
prevalent depression	152,279	Fixed effect: 1.08 (1.04; 1.13)
		Random effect: 1.07 (0.94; 1.23)
incident depression	89,459	Fixed effect: 1.08 (1.01; 1.15)
		Random effect: 1.18 (1.02; 1.35)
quality score ≥ 8	219,389	Fixed effect: 1.10 (1.05; 1.14)
		Random effect: 1.14 (1.02; 1.26)
only women	91,470	Fixed effect: 1.03 (0.99; 1.08)
		Random effect: 1.02 (0.91; 1.14)

## Supplementary Materials

The following are available online at https://www.mdpi.com/1660-4601/17/18/6686/s1, Table S1: Search strategy in PubMed/MEDLINE.
