# Supplementary material for: Red and Processed Meat Consumption and Risk of Depression: A Systematic Review and Meta-Analysis
Source: Int J Environ Res Public Health. 2020 Sep 14;17(18):6686. doi: 10.3390/ijerph17186686 (PMC7559491; doi:10.3390/ijerph17186686)
Supplement: Supplementary file 1 [file ijerph-17-06686-s001.docx]

**Supplementary Table S1.** Search strategy in PubMed/MEDLINE.

| **SET** | **PubMed/Medline** |
| --- | --- |
| 1  2  3  4  5  6  7  8  9  10  11  12  13  14 | "processed meat"[Title/Abstract]  "processed meat"[Text Word]  "processed meat"  "Red Meat"[Mesh]  "red meat"[Title/Abstract]  "red meat"[Text Word]  "red meat"  meat[Title/Abstract]  meat[Text Word]  meat  "Diet, Western"[Mesh]  "Diet, Paleolithic"[Mesh]  "Animal Proteins, Dietary"[Mesh]  "Meat"[Mesh] |
| 15 | Sets 1-14 were combined with “OR” |
| 16  17  18  19 | "Depressive Disorder"[Mesh]  "Depression"[Mesh]  depression[Title/Abstract]  depressive[Title/Abstract] |
| 20 | Sets 16-19 were combined with “OR” |
| 21 | Sets 15 and 20 were combined with “AND” |
